# Prioritizing Endangered Species in Genome Sequencing: Conservation Genomics in Action with the First Platinum-Standard Reference-Quality Genome of the Critically Endangered European Mink *Mustela lutreola* L., 1761

**DOI:** 10.3390/ijms241914816

**Published:** 2023-10-01

**Authors:** Jakub Skorupski, Florian Brandes, Christian Seebass, Wolfgang Festl, Przemysław Śmietana, Jennifer Balacco, Nivesh Jain, Tatiana Tilley, Linelle Abueg, Jonathan Wood, Ying Sims, Giulio Formenti, Olivier Fedrigo, Erich D. Jarvis

**Affiliations:** 1 Institute of Marine and Environmental Sciences, University of Szczecin, Wąska 13 St., 71-415 Szczecin, Poland; 2 Polish Society for Conservation Genetics LUTREOLA, Maciejkowa 21 St., 71-784 Szczecin, Poland; 3 Wildtier- und Artenschutzstation e.V., Hohe Warte 1, 31553 Sachsenhagen, Germany; 4 EuroNerz e.V., Kleine Gildewart 3, 49074 Osnabrück, Germany; 5 Vertebrate Genome Laboratory, The Rockefeller University, 1230 York Avenue, Box 366, New York, NY 10065, USA

**Keywords:** conservation genomics, critically endangered species, European mink, European Mink Centre, *Mustela lutreola*, platinum-quality genome, reference genome, whole-genome sequencing

## Abstract

The European mink *Mustela lutreola* (Mustelidae) ranks among the most endangered mammalian species globally, experiencing a rapid and severe decline in population size, density, and distribution. Given the critical need for effective conservation strategies, understanding its genomic characteristics becomes paramount. To address this challenge, the platinum-quality, chromosome-level reference genome assembly for the European mink was successfully generated under the project of the European Mink Centre consortium. Leveraging PacBio HiFi long reads, we obtained a 2586.3 Mbp genome comprising 25 scaffolds, with an N50 length of 154.1 Mbp. Through Hi-C data, we clustered and ordered the majority of the assembly (>99.9%) into 20 chromosomal pseudomolecules, including heterosomes, ranging from 6.8 to 290.1 Mbp. The newly sequenced genome displays a GC base content of 41.9%. Additionally, we successfully assembled the complete mitochondrial genome, spanning 16.6 kbp in length. The assembly achieved a BUSCO (Benchmarking Universal Single-Copy Orthologs) completeness score of 98.2%. This high-quality reference genome serves as a valuable genomic resource for future population genomics studies concerning the European mink and related taxa. Furthermore, the newly assembled genome holds significant potential in addressing key conservation challenges faced by *M. lutreola*. Its applications encompass potential revision of management units, assessment of captive breeding impacts, resolution of phylogeographic questions, and facilitation of monitoring and evaluating the efficiency and effectiveness of dedicated conservation strategies for the European mink. This species serves as an example that highlights the paramount importance of prioritizing endangered species in genome sequencing projects due to the race against time, which necessitates the comprehensive exploration and characterization of their genomic resources before their populations face extinction.

## 1. Introduction

**Figure 1:**
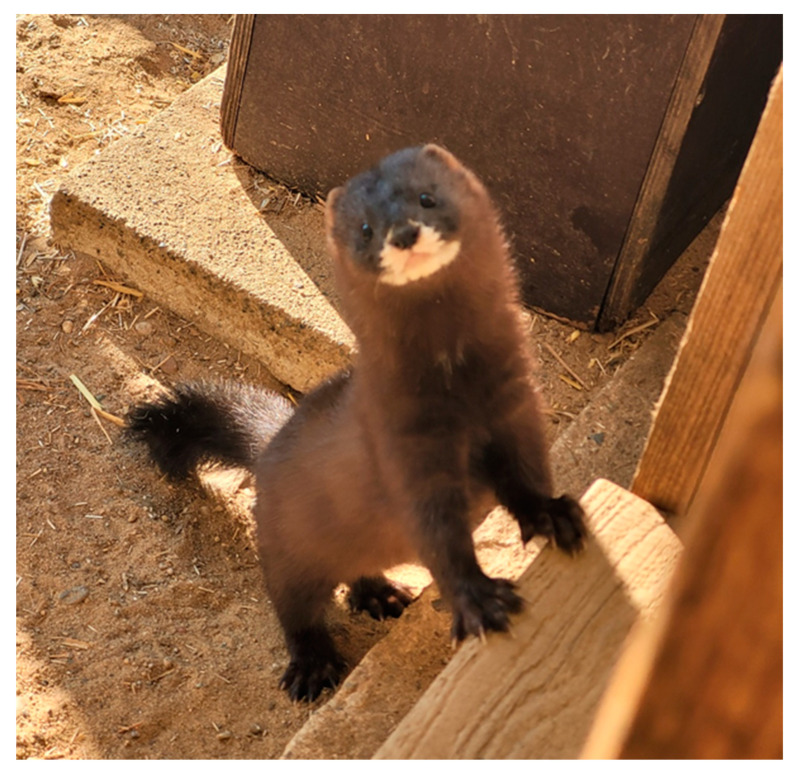
European mink male in the captive breeding facility of the Wildtier- und Artenschutzstation e.V. in Sachsenhagen, Germany (Author: Jakub Skorupski).

The alarming decline of biodiversity worldwide necessitates urgent conservation measures, particularly for wild, endangered, and understudied species. According to the International Union for Conservation of Nature’s (IUCN) Red List of Threatened Species, of the 5973 mammal species assessed, 1340 were classified as threatened with extinction, including 233 critically endangered taxa [1]. This number includes wild, non-domesticated, non-model species, whose rapidly vanishing genetic resources may never be adequately explored and described. This concerns the European mink *Mustela lutreola* L., 1761, a critically endangered, semiaquatic, secretive, and solitary representative of the mustelid family (Eukaryota; Metazoa; Chordata; Craniata; Vertebrata; Euteleostomi; Mammalia; Eutheria; Laurasiatheria; Carnivora; Caniformia; Mustelidae; Mustelinae; *Mustela*) [2,3] (Figure 1). The species is listed in the Annex II to the Bern Convention on the Conservation of European Wildlife and Natural Habitats, the Annexes II and IV (priority species) of the Council Directive 92/43/EEC on the conservation of natural habitats and of wild fauna and flora, and in the Carpathian List of Endangered Species (critically endangered species), and considered one of the most endangered mammalian species in the world [4,5].

**Figure 2:**
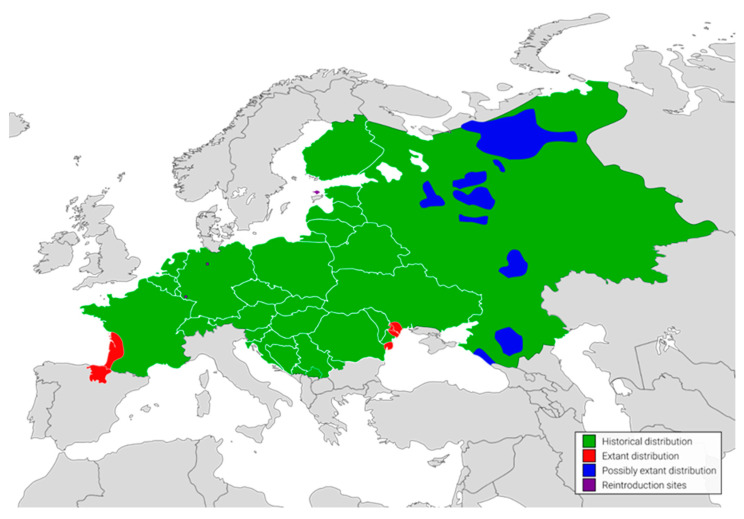
Historical and current European mink range in Europe (Author: Zygmunt Horodyski).

By the 19th century, European mink populations were relatively abundant and distributed across freshwater habitats throughout a large part of continental Europe [5] (Figure 2). Following a combination of habitat loss and fragmentation, commercial over-hunting for fur and the effects of introduced, invasive American mink *Neogale vison* Schreber, 1777 lead to a dramatic depletion of the species’ populations, in terms of both shrinkage of a geographical range by 97%, and a reduction in the number of individuals persisting in the wild to about 5000 [5,6,7]. What is more, an expected decline rate in number over the next three generations exceeds 80% [5]. These days, only three isolated, declining populations, restricted to the European part of Russia, the Danube Delta, south-western France and north-eastern Spain survived [5]. Reintroduction efforts successfully established populations in Estonia on Hiiumaa Island, as well as in Germany at two locations in Saarland and Lower Saxony [5].

In response to the critical status of *M. lutreola*, conservation efforts have been initiated in the late 20th century, focusing on captive breeding programmes (i.e., the European Association of Zoos and Aquaria (EAZA) Ex-situ Programme (EEP) for European mink, established in 1992, regional captive breeding programme initiated in Spain in 2004, and the European Mink Breeding Centre of the Ilmen Nature Reserve in Russia, operating since 2010), habitat restoration, local reintroduction initiatives (launched in Estonia, France, Germany, Russia and Spain), reintroduced populations monitoring, and public awareness campaigns [8]. Of key importance for the effectiveness and efficiency of these measures are genomic research, informing targeted and evidence-based conservation strategies [9,10]. By studying the genomic makeup of the European mink, valuable insights into its population structure, genetic diversity, and evolutionary history can be gained. This knowledge is essential for understanding the unique adaptations and vulnerabilities of this species, as well as identifying and quantifying inbreeding, hybridization, and introgression [11,12,13,14,15]. Such information is vital for developing targeted conservation efforts, including captive breeding programmes, reintroduction and translocation strategies, and genetic management plans, to halt ongoing depletion of genetic diversity of *M. lutreola* sparse, local populations, and preserve their genetic integrity and long-term viability, ultimately aiding in their survival and recovery in the wild [8].

Comprehensive research of the genetic resources of a critically endangered species is of paramount importance from both cognitive and practical perspectives. Not only do we gain new knowledge, enable a deeper understanding of its biology and conservation needs, but we can also provide critical information for informed conservation decision-making. This knowledge is invaluable for collecting and preserving irreplaceable genetic heritage that is on the verge of being lost forever and unnoticed, particularly in the case of a non-charismatic, rare, and elusive species. Meanwhile, despite the alarming situation of the European mink, the number of studies on its genetics and genomics is limited and urgently need to be completed [8]. To date, there has not been a reference genome for the species, its mitochondrial genome was only sequenced in 2022, and no data were stored in the Sequence Read Archive (SRA) in the GenBank [8,16].

To address this problem, we present for the first time a platinum-standard reference-quality genome, i.e., high confidence, contiguous, de novo assembly of the haplotype-resolved diploid genome with full chromosome scaffold, for the European mink. By sharing this valuable resource, we aim to shed light on the species genomics, facilitate future research on its evolutionary history and present population structure, dynamics and adaptation, and thus catalyze global efforts towards the conservation and management of *M. lutreola* and other threatened mustelids, by offering novel perspectives on conservation genomics of this taxa.

## 2. Results

**Figure 3:**
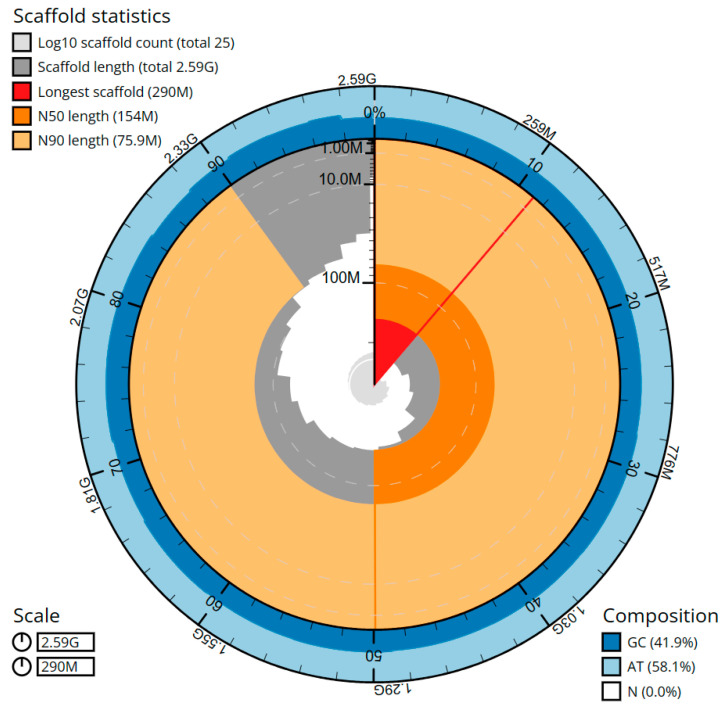
Snail plot summary (the BlobToolKit Snailplot) of the *Mustela lutreola* (mMusLut2) genome assembly (the primary plot is partitioned into 1000 size-ordered bins distributed along the circumference; the scaffold length distribution is presented in dark grey, with the plot’s radius adjusted to the longest scaffold (shown in red); additionally, two arcs in orange and pale-orange represent scaffold N50 and N90, respectively; the cumulative scaffold count is illustrated on a logarithmic scale, depicted by the pale grey spiral, and white scale lines indicate successive orders of magnitude; surrounding the inner plot, the blue and pale-blue areas indicate the GC, AT, and N content in the same bins).

The cumulative length of the final assembly amounts to 2586.27 Mbp, including 39 gaps and encompassing 64 sequence contigs and 25 scaffolds, with a contig N50 (LG = 11) and a scaffold N50 (LG = 7) of 83.36 Mbp and 154.08 Mbp, respectively (Figure 3, Table 1). The presence of the 11 largest contigs containing 50% of the genomic sequences demonstrates significant contiguity within the assembly. On average, 36.15× coverage for the PacBio sequencing and 66.85× for the Hi-C sequencing was achieved. The genome assembly exhibited an average contig length of 40.41 Mbp, representing the mean size of the individual DNA fragments prior to scaffolding. The average scaffold length was 103.45 Mbp, indicating the typical size of the contiguous DNA sequences generated. The total gap length in the scaffolds amounted to 7.8 kbp, with an average gap length of 200 bp. Through manual assembly curation, a total of 27 missing or missed joins were rectified, and no sequence removed as haplotypic duplication, resulting in a 26.5% drop in the scaffold number. The scaffold N50 value remained unaltered, while a minor increase of 0.25% in the N90 length was observed as a result of the curation process.

**Figure 4:**
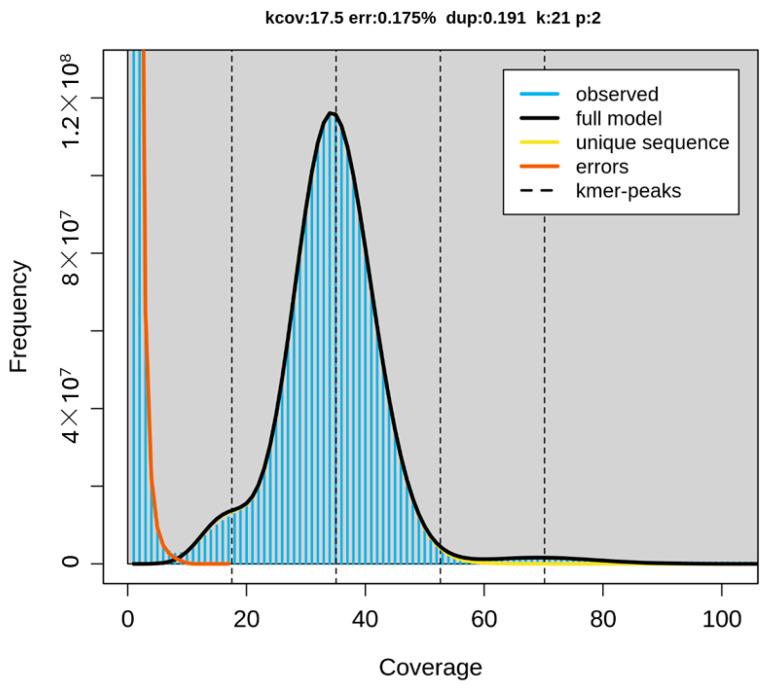
GenomeScope k-mer profile plot of the European mink dataset (mMusLut2), showing model fit (black) and observed k-mer frequencies (blue), with a distinct peak of very high frequency k-mers of highly enriched organelle sequences (Kcov—mean k-mer coverage, err—estimated error rate of the reads, dup—average rate of read duplications, k—k-mer size used, p—ploidy level).

**Figure 5:**
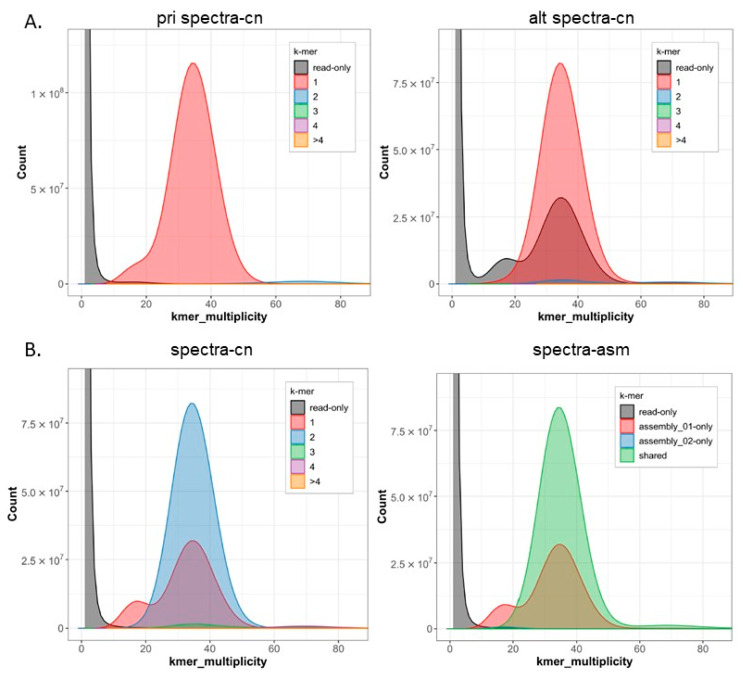
Merqury spectrum plots for haploid assemblies of a *Mustela lutreola* (mMusLut2) genome (pri/01—primary assembly, alt/02—alternate assembly): (**A**). Copy number spectra (spectra-cn) of the k-mers collected from Illumina reads, (**B**). Contigs (spectra-cn) and assembly (spectra-asm) spectrum plot for evaluating k-mer completeness (k-mers colored by their presence in the reads and primary/alternate assemblies).

The newly sequenced genome displayed a GC base content of 41.85%, with adenine, cytosine, guanine, and thymine nucleotides accounting for 29.08%, 20.94%, 20.91%, and 29.07%, respectively. Repeat sequences constitute approximately 24.37% of the genome. The genome assembly analysis revealed that 99.83% of the regions were homozygous, while the remaining 0.17% were heterozygous (Figure 4 and Figure 5).

The estimated Quality Value (QV) of the Hifiasm assembly was 64.34, with k-mer completeness of 99.17%, and error rate of 3.6826 × 10^−7^. The final, purged assembly had a BUSCO completeness score (C) of 98.15%. The score breakdown indicates that 95.92% of the expected complete single-copy genes (S) were identified as complete sequences, while 2.24% of the single-copy genes were found as duplicates or fragmented sequences (D). The fragmented fraction (F) score was 0.86%, denoting a proportion of expected complete duplicated genes found in the assembly. The missing (M) score, indicating a rate of missing expected complete genes, was 0.98%. Regarding false duplications, the BUSCO results showed promising outcomes, and the primary-only spectra plot from Merqury displayed a clean pattern. The purged assembly demonstrated completeness of 99.13%, a QV of 64.44, and an error rate of 3.6005 × 10^−7^. Detailed characteristics of pre- and curated genome assemblies of a *M. lutreola* are presented in Table 2.

**Figure 6:**
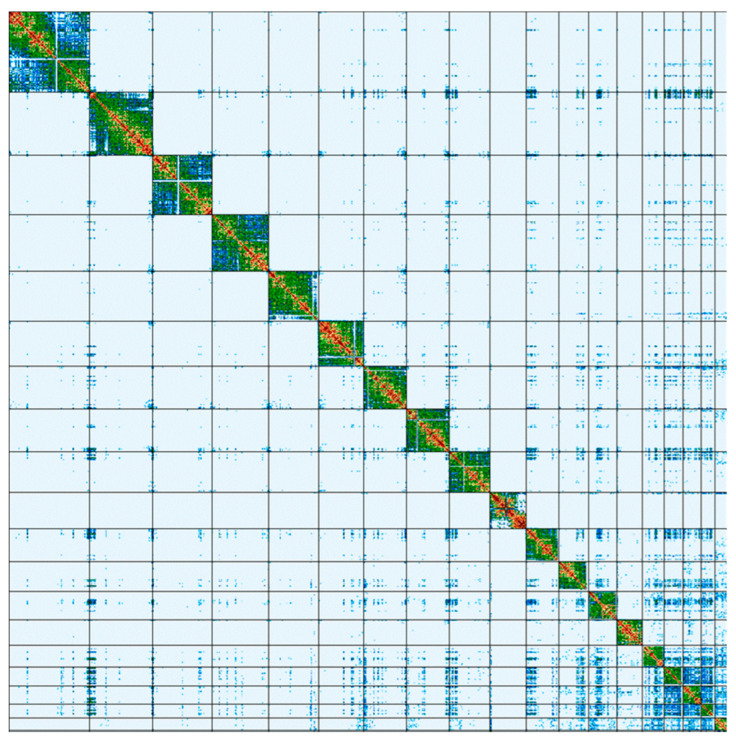
Hi-C contact map of the mMusLut2 assembly, visualized in PretextMap. Scaffolds representing chromosomes are ordered by size from top left (largest) to bottom right (smallest). The color block demonstrates the intensity of the interaction from blue (low) to red (high).

The vast majority (99.9985%) of the genome assembly was assigned to 20 C-scaffolds (pseudomolecules) [17], comprising 18 autosomes, and the X and Y sex chromosomes (Figure 6). Additionally, one unplaced (without a chromosome assignment) and four unlocalized (not localized to a specific position in the chromosome) scaffolds were identified in the assembly. Chromosome-scale scaffolds, confirmed by the Hi-C data, are named in order of size and characterized in Table 3.

The complete mitochondrial genome assembly is 16,552 bp in length, and displayed a level of identity ranging from 99.62% to 99.94% with previously sequenced mitochondrial genomes of *M. lutreola*, deposited in the GenBank [16].

Metadata for spectral estimates, sequencing runs, contaminants, and pre-curation, and curated assembly statistics are available at https://genomeark.s3.amazonaws.com/index.html?prefix=species/Mustela_lutreola (accesed on 1 July 2023).

## 3. Discussion

The genome sequencing of various mustelids has provided valuable insights into their genetic composition and evolutionary history. Out of the 67 species in the Mustelidae family, 17 have reference genome assemblies ready and deposited in GenBank, while only in the case of seven species has a chromosome-level assembly been achieved (Table 4) [18,19]. European mink stands as the only critically endangered species within this group that has now attained a reference genome of a platinum quality. A platinum genome is defined as a high-quality, near error-free and gapless, chromosome-level, haplotype-phased, reference genome assembly [20,21]. Additionally, the following standards were drawn for the Vertebrate Genomes Project of the Genome 10K consortium: N50 size of at least 1 Mbp for contigs and 10 Mbp for scaffolds, sequence error frequency of up to 1 in 10,000 bp, structural variants confirmed by multiple technologies, at least 90% of the sequence assigned to chromosomes and haplotype-phased [21,22]. Furthermore, a standard VGP reference genome involves an automated workflow that combines long-read sequencing, linked-read sequencing, optical mapping, and Hi-C data, with a final manual curation step to enhance the genome assembly and minimize errors [20,23]. The genome sequence reported in this article was assembled following the VGP quality requirements and meets the abovementioned conditions.

The size of the reported genome of a *M. lutreola* fits well with previously sequenced genomes of other representatives of the Mustela genus, being the biggest among the so-called ferret group, clustering the European polecat, the steppe polecat, the black-footed ferret, and the European mink [16,24,25]. The European mink genome assembly was found to be noticeably close in size and the GC-content to the earlier estimates, which predicted the genome size of the species to be around 2.411–2.474 Gbp and the GC content to be approximately 42%, based on the sequenced genomes of the ferret (MusPutFur1.0, GenBank assembly accession: GCF_000215625.1) and the European polecat (polecat_10x_lmp_bionano, GenBank assembly accession: GCA_902207235.1) [16].

The number of whole-chromosome pseudomolecules assembled in the European mink’s reference genome (18 autosomes and two heterosomes) is consistent with a diploid chromosome number reported for this species (2*n* = 38) [26]. Such a diploid number of chromosomes is typical of the family Mustelidae, occurring in over 60% of its representatives [27].

The high-quality reference genome of European mink represents the fulfilment of earlier calls for whole-genome sequencing of this critically endangered species [8,28], marking a significant step forward in advancing our understanding of the species’ genetic composition and promising to enhance conservation efforts. A platinum quality reference genome serves as a highly accurate and reliable resource for various research applications, including evolutionary studies, population genomics, comparative genomics and functional genomics [13,29]. Such reference genomes are particularly valuable for species of conservation concern, where accurate genomic information is crucial for effective conservation efforts and understanding the species’ biology [10,13]. The significant application potential of genomics in addressing conservation problems is well-documented for various carnivorans, e.g., African wild dog *Lycaon pictus*, Eastern wolf *Canis lupus lycaon*, puma *Puma concolor*, Iberian lynx *Lynx pardinus*, and wolverine *Gulo gulo* [11,30,31,32].

The reference genome of the European mink can play a pivotal role in addressing the conservation problems faced by this species, enabling the revision of management units to effectively manage genetic diversity within populations and minimize the outbreeding risk associated with inter-population translocations, providing comprehensive insights into the impacts of captive breeding, resolving phylogeographic questions, and facilitating the evaluation of conservation program efficiency and effectiveness [8,33]. It is important to recognize that the application of genomics to conservation often encounters a significant challenge—the high costs associated with molecular analyses. However, this is where advanced genomic tools come into play. Through techniques like whole-genome re-sequencing and reduced-representation approaches, conservation genomics offers a potential solution to mitigate these cost constraints, as these methodologies can reduce the number of markers required for analysis and monitoring projects in the field of conservation activities [11,12,13,14,15,33].

Comprehensive population genomics studies are crucial, as the limited data on interpopulation genetic diversity could significantly impede the effectiveness of captive breeding, reintroduction programs, and potential translocations for persisting wild populations of *M. lutreola* [28,34,35,36,37]. By analysing the genetic variation, relatedness between populations, and identifying adaptive traits loci, it aids in the establishment of appropriate management units for targeted conservation. One prominent example of the application of population genomics is the ongoing debate surrounding the potential inclusion of the Spanish conservation breeding initiative in the European Endangered Species Programme (EEP) for European mink [28]. Additionally, plans to obtain new founders from the wild Romanian population emphasize the importance of understanding historical population dynamics and connectivity between existing European mink populations [14,28].

Compared to traditional genetic approaches, the reference genome provides advanced genomic tools to investigate evolutionary relationships among different European mink populations. It helps in determining historical patterns of migration and divergence, shedding light on the species’ phylogenomic and phylogeographic history [38,39]. Such understanding is essential for re-evaluating Evolutionarily Significant Units (ESUs) and Management Units (MUs) for *M. lutreola* [34,40,41,42]. These units guide conservation actions based on distinct evolutionary lineages and aim to preserve the genetic diversity of the species [40,41]. Moreover, the reference genome of the European mink serves as a valuable tool in resolving uncertainties about its past distribution over continental Europe [43,44]. It helps identify regions of high genetic diversity, indicating historical refugia or areas of long-term stability for the species, as well as regions of genetic variation associated with adaptation to specific environments, habitat use, disease resistance, responses to changing conditions, or other crucial ecological factors [10,29]. Furthermore, it can provide insights into the evolutionary history of the species and its relationship with other mustelids.

Another pressing concern in the European mink conservation revolves around the assessment of the impact of the conservation breeding process on the development of traits essential for survival in the wild, specifically focusing on the adaptation to captivity [8]. Farquharson et al. [45] reported strong effects of inbreeding on the European mink offspring fitness in the EEP captive breeding program, highlighting the importance of addressing genetic management. One of the factors that reduces reproductive success in captivity and reconstituted (reintroduced) populations is aggressive behaviour exhibited by males toward females, which can lead to their exclusion from mating [46,47]. However, without a clear understanding of the heritability of these personality traits, assessing the risk of reducing genetic variation in reintroduced populations due to the release of individuals with specific personality types becomes challenging [46,47,48]. Furthermore, the reference genome can help to identify regions of the genome that are associated with local adaptation or specific traits relevant to survival in the wild [10,29]. This information can guide breeding strategies to ensure that valuable adaptive and survival traits are retained in captive populations, even when introducing new individuals to counteract inbreeding. By avoiding excessive inbreeding, the fitness and resilience of an offspring can be improved, increasing their chances of survival in both captive and future reintroduction settings.

Genomic information is essential to examine the impact of reintroduced individuals on shaping the gene pools of wild populations to address potential issues of outbreeding and the risk of losing unique adaptations [40,43]. In this regard, genomic-scale analyses serve as a valuable tool in evaluating potential fitness losses, thus facilitating more informed decisions and enhancing the success of reintroduction and translocation efforts [10,13]. The reference genome can support conservation breeding strategies by assisting in the selection of founders for captive breeding programs. It also helps optimize breeding pairs and prevents over-representation of certain lineages, ensuring that individuals chosen for reproduction possess optimal genetic diversity and reduce the risk of inbreeding depression [10,13].

With the reference genome, it becomes feasible to conduct genome-wide monitoring of the European mink local populations. This allows researchers to track changes in genetic diversity, detect potential threats to specific populations, and evaluate the effectiveness of conservation interventions over time, across multiple generations. Understanding the genetic variation within and between populations can help identify populations with low genetic diversity, which may be at greater risk of inbreeding depression and reduced adaptive potential, as well as genetically unique and vulnerable populations. Conservation efforts can then prioritize these populations for targeted management and genetic supplementation if necessary (genetic rescue) [49]. Genome-scale monitoring provides real-time information on the genetic health and status of endangered species populations, enabling adaptive management strategies to ensure their long-term survival, recovery, and genetic health [50]. Adaptive management is a dynamic and flexible approach characterized by continuous revisions of conservation strategies based on genetic data and observed fitness outcomes, allowing for timely adjustments and improvements to enhance conservation effectiveness.

In conclusion, embracing reference genome offers a powerful and comprehensive approach to address the conservation issues faced by *M. lutreola*, enhancing the prospects for its preservation and sustainable recovery. Future research directions in the European mink conservation genomics, based on the reported genome assembly, may involve building a reference pangenome to enable detailed population genomic studies, monitoring interpopulation genetic diversity patterns using restriction-site associated DNA sequencing (RADseq, ddRAD), and conducting a genome-wide scan for runs of homozygosity (ROH) to detect signatures of selection and estimate inbreeding [15,29,51]. Many of the conservation issues observed in this species could have been prevented if decisions regarding the necessary actions were not made before obtaining knowledge about its genome, but resulted from it.

The reported genome assembly also provides perspectives for planning, implementing, monitoring, and evaluating conservation interventions for other closely related taxa. The reference genome of the European mink can serve as a valuable reference for conducting reference-based assembly or designing primers for targeted sequencing of specific genomic locations in mustelids, whose genomes are yet to be fully revealed (e.g., *Mustela eversmanii*, *Mustela sibirica*, *Mustela itatsi*).

In the context of large genome sequencing programs, the prioritization of species sequencing order becomes crucial, as it enables the optimization of funding allocation, research interest, and workload alignment, ensuring that endangered species receive the necessary resources and attention commensurate with their conservation urgency. By prioritizing the comprehensive research of genomic resources in critically endangered species like the European mink, we not only gain valuable knowledge for conservation decision-making, but also preserve the irreplaceable genetic heritage that is on the brink of being lost forever.

The relatively low research interest in the European mink is incongruent with its critical situation. This highlights the significance of promotional and informational campaigns aimed at drawing attention and raising awareness in society about its alarming threat of extinction, ultimately ensuring adequate focus and attention of the scientific community towards understanding and conserving this fascinating species, deserving effective protection.

## 4. Materials and Methods

### 4.1. Sample Collection and DNA Extraction

The European Mink Genome Project (https://www.ncbi.nlm.nih.gov/bioproject/986837 (accesed on 1 July 2023)) was initiated in October 2020 by the consortium of the European Mink Centre (Szczecin, Poland, http://europeanminkcentre.com/ (accesed on 1 July 2023)), the Vertebrate Genome Laboratory of the Rockefeller University (New York, USA, https://www.vertebrategenomelab.org/ (accesed on 1 July 2023)), the Wildtier- und Artenschutzstation e.V. association (Sachsenhagen, Germany, https://wildtierstation.de/ (accesed on 1 July 2023)), and the EuroNerz e.V. association (Osnabrück, Germany, https://www.euronerz.de/ (accesed on 1 July 2023)), led by the University of Szczecin (Szczecin, Poland). The assembly was also contributed to by the Vertebrate Genomes Project (VGP), whose assembly pipeline (v. 2.0) and style quality metrics were implemented and followed [21,52,53,54].

Samples were taken from two (mMusLut2 and mMusLut3) captive-born, adult, clinically healthy, closely related *M. lutreola* males (heterogametic sex), during a routine veterinary examination, in accordance with the principles of animal welfare. Sampled individuals are kept by the European mink conservation breeding facility of the Wildtier- und Artenschutzstation e.V. (Sachsenhagen, Lower Saxony, Germany; 52°23′51″ N 9°12′58″ E), participating in the EAZA EEP for European mink, and are characterized in the Table 5. Both sampled individuals presented phenotypic features typical for the species [2]. The samples were collected by Florian Brandes (veterinarian, the Wildtier- und Artenschutzstation e.V.), assisted by Jakub Skorupski and Przemysław Śmietana (both University of Szczecin), on 14 March 2022 in the Wildtier- und Artenschutzstation e.V. headquarters. Individual mMusLut2 (specimen M1207) was used for acquisition of the genome sequence and HiFi data generation, while individual mMustLut3 (specimen M1287) was used for scaffolding using Hi-C.

Whole blood samples were sterile collected by a cephalic vein venipuncture under inhalant anaesthesia [55,56]. Approximately 0.75 mL of blood per individual was drawn into the BD Vacutainer^®^ tube with K2 EDTA (Becton, Dickinson and Company, New Jersey, NJ, USA). The samples were immediately gently mixed by inverting the tubes 8–10 times to ensure proper anticoagulation and prevent clot formation prior to further laboratory analysis.

After collection, the samples were snap-frozen at −80 °C to preserve the integrity of the biological material. The frozen tubes were packed in insulated containers with at least 10 kg of dry ice and shipped overnight to the Vertebrate Genomes Laboratory (VGL) at the Rockefeller University (New York, NY, USA), for further analyses.

Ultra-high molecular weight (UHMW) DNA was extracted from frozen samples using the Nanobind^®^ Bionano Prep SP Dna isolation kit (Bionano Genomics Inc., San Diego, CA, USA) method, according to the manufacturer’s protocol. The Nanobind^®^ magnetic disk applied, enabled automatic lysis, binding, washing and elution, and efficiently minimized fragmentation and preserved long DNA molecules required for accurate long-read sequencing.

### 4.2. Sequencing

To achieve an optimal insert size for the PacBio sequencing, the Megaruptor^®^ 3 system (Diagenode Inc., Denville, NY, USA) with a standard hydropore and speed setting 28 was used to shear the UHMW DNA. The fragmented DNA was sheared to an average size of 17,000 bp, suitable for highly accurate long-read sequencing. Sheared DNA was concentrated and cleaned using 0.45× Ampure PB (Pacific Biosciences of California Inc., Menlo Park, CA, USA). The concentration and length of the purified sheared DNA were evaluated with the QubitTM 3.0 fluorometer (Thermo Fisher Scientific Inc., Waltham, MA, USA) and the Femto Pulse system (Agilent, Santa Clara, CA, USA).

The PacBio HiFi (high-fidelity) circular consensus sequencing library was prepared using PacBio’s 3.0 template preparation kit (Pacific Biosciences of California Inc., Menlo Park, CA, USA), following the manufacturer’s protocol. With a single-molecule read accuracy surpassing 99.9%, the PacBio platform produces HiFi reads using circular consensus sequencing (CCS) mode on PacBio long-read systems, spanning an optimal range between 15 kb and 20 kb. An input mass of 5 µg of sheared DNA was used for library construction. The library was size-selected using the Pippin HTTM (Sage Science Inc., Beverly, MA, USA) automated, high throughput gel-based method to obtain the desired insert size. HiFi data were generated for the mMusLut2 (paternal) individual. The Cutadapt v. 4.4 algorithm was used to remove the reads found to have an adapter inside of it [57].

The prepared library was sequenced on the Sequel IIe system using PacBio’s binding kit 3.2 and sequencing plate 2.0 (Pacific Biosciences of California Inc., Menlo Park, CA, USA). A total of six 8M SMRT (single-molecule real-time) cells were used to generate long-read sequencing data [58].

The high-throughput chromosome conformation capture (Hi-C) sequencing data were generated from the blood sample of the mMustLut3 (filial) individual applying the Dovetail™ Hi-C kit (Dovetail Genomics LLC, Scotts Valley, CA, USA) and sequenced on NovaSeq 6000 instrument (Illumina Inc., San Diego, CA, USA), following the manufacturer’s instructions.

### 4.3. De Novo Genome Assembly, Curation, and Quality Control

The long-read sequencing data were processed using PacBio’s SMRT Analysis software v. 11.1 pipeline (Pacific Biosciences of California Inc., Menlo Park, CA, USA) to obtain high-quality long reads. The Hifiasm (version 0.18.8+galaxy1) assembler, specifically developed for PacBio HiFi reads, was used to generate the continuous and complete de novo assembly [59]. Pseudohaplotype assemblies (primary/principal and alternate) of mMusLut2 were scaffolded using the haplotype-specific Hi-C reads of mMusLut3, using both Hifiasm and YaHS version 1.2a.2+galaxy0 [60] tools. The abovementioned primary assembly, encompassing homozygous and one set of loci for heterozygous regions, represents a more complete representation of an individual’s genome and is preferred for downstream analyses as it provides both homozygous and heterozygous regions. The alternate assembly, or haplotigs, includes the alternate loci from the other haplotype’s heterozygous regions, and being less complete than the primary assembly, it lacks representation of homozygous regions [54].

The Hi-C analysis was performed using the Arima-HiC kit 2 (Arima Genomics Inc., Carlsbad, CA, USA). Utilizing chromosome conformation Hi-C data, the primary assembly contigs were organized and linked into larger scaffolds [61]. The Hi-C reads were aligned to the genome assembly to generate a contact map, visualized using the PretextView v. 0.2.5 (https://github.com/wtsi-hpag/PretextView (accessed on 1 June 2023)).

By decomposing the sequencing data into k-length substrings, counting the occurrence of each k-mer, and determining its frequency, Meryl v. 1.4 enabled the generation of the k-mer profile [62]. Genome properties, including genome size, repetitiveness, and heterozygosity rates, were inferred from the k-mer histogram generated by Meryl using GenomeScope v. 2.0, a tool that utilizes sequencing reads using a kmer-based statistical approach [63]. The expected genome size was computed from the k-mer genome coverage. K-mers were also used for initial, reference-free genome profiling.

The mitochondrial genome was assembled from PacBio High Fidelity reads, with the MitoHiFi v. 3.2 pipeline [64]. The NCBI Reference Sequence NC_056132.1 was used as a reference. The comparison with previously sequenced complete mitogenome sequences of *M. lutreola*, deposited in the GenBank (Accession No. NC_056132.1, MW197426.1, MW197425.1, MW197424.1, MT304869.1, MW197423.1), was conducted using BLASTN v. 2.14.1+ programme (Nucleotide collection, megablast option) [65].

The gfastats v. 1.3.6 [66] was applied to check for contamination and correct the assemblies at each assembly stage. The Merqury v. 1.3 platform [62] was used to perform quality control at the contiging and purging stages (estimation of the consensus Quality Value (QV) scores of the final assembly and k-mer completeness). The gfastats v. 1.3.6 tool [66] was also used to compute the number of scaffolds, average of scaffold lengths, number of gaps, L50, N50, and total assembled bases.

The BUSCO (Benchmarking Universal Single-Copy Orthologs) scores, assessing the completeness and quality of genome assemblies by evaluating the presence and integrity of a set of highly conserved genes that are expected to be present as single copies in a given genome (vertebrata_odb10 dataset, *n* = 3354), were granted within the BUSCO v. 5.3.2 software, applying the Metaeuk 6.a5d39d9 and HMMsearch v. 3.1 gene predictor algorithms [67,68]. To improve overall assembly quality, gap filling, removing false duplications, collapsed repeats and very low coverage regions, as well as haplotigs purging was performed using the purge_dups v. 1.2.5+galaxy4 package [69], based on read depth. Parameters derived from the GenomeScope output were used to define cutoffs.

Manual curation of final assembly was performed using HiGlass v. 1.11 [70] and PretextMap v. 0.1.9 [71] tools, to resolve potential mis-assemblies, missed joins, duplications and collapses, and remove any contaminants.

### 4.4. Genome Annotation and Data Availability

This Whole Genome Shotgun project has been deposited at DDBJ/ENA/GenBank under the accession JAUCGO000000000 (JAUCGO010000001–JAUCGO010000026) and JAUCGP000000000 (JAUCGP010000001–JAUCGP010012043) and will be subjected to comprehensive annotation by the National Centre for Biotechnology Information (NCBI) refseq Eukaryotic Genome Annotation Pipeline [72]. Thus, the first platinum-standard reference-quality genome sequence is released openly for reuse—accession information of raw sequence data and the final assembled genome are given in Table 6.

## Figures and Tables

**Table 1:** Overall contigs and scaffolds characteristics for the primary and alternate assemblies of mMusLut2.

NG	Primary Assembly				Alternate Assembly			
	Contigs		Scaffolds		Contigs		Scaffolds	
	LG	Length	LG	Length	LG	Length	LG	Length
10	2	161.66 Mbp	1	290.10 Mbp	137	0.79 Mbp	137	0.79 Mbp
20	4	146.10 Mbp	3	211.29 Mbp	417	0.53 Mbp	417	0.53 Mbp
30	6	117.74 Mbp	4	202.57 Mbp	811	398.20 Kbp	811	398.20 Kbp
40	8	94.97 Mbp	5	177.47 Mbp	1321	311.74 Kbp	1321	311.74 Kbp
50	11	83.36 Mbp	7	154.08 Mbp	1968	245.42 Kbp	1968	245.42 Kbp
60	14	72.93 Mbp	8	151.51 Mbp	2788	193.83 Kbp	2788	193.83 Kbp
70	18	63.47 Mbp	10	133.08 Mbp	3841	148.62 Kbp	3841	148.62 Kbp
80	23	42.37 Mbp	12	104.55 Mbp	5262	105.12 Kbp	5262	105.12 Kbp
90	31	25.02 Mbp	15	75.87 Mbp	7424	64.08 Kbp	7424	64.08 Kbp
100	64	38.84 Kbp	25	38.84 Kbp	12,043	10.04 Kbp	12,043	10.04 Kbp
1.000x	64	2.59 Gbp	25	2.59 Gbp	137	0.79 Mbp	137	0.79 Mbp

NG(X) is the length for which the collection of all contigs/scaffolds of that length or longer covers at least X% of the assembled genome, while LG(X) is the number of contigs/scaffolds equal to or longer than NG(X). NG based on genome size 2.59 Gbp.

**Table 2:** Characteristics of the *Mustela lutreola* genome assembly (mMusLut2).

Feature	Pre-Curation Assembly	Curated Assembly
Expected genome size	2,572,597,696	2,586,268,927
Number of scaffolds	34	25
Total scaffolds length	2,586,267,127	2,586,268,927
Average scaffold length	76,066,680.21	103,450,757.08
Scaffold N50	154,078,643	154,078,643
Scaffold auN	163,788,738.52	165,291,595.19
Scaffold L50	7	7
Largest Scaffold	289,168,877	290,104,894
Smallest scaffold	38,844	38,844
Number of contigs	64	64
Total contig length	2,586,261,127	2,586,261,127
Average contig length	40,410,330.11	40,410,330.11
Contig N50	83,356,672	83,356,672
Contig auN	90,760,552.39	90,760,552.39
Contig L50	11	11
Largest contig	171,611,272	171,611,272
Smallest contig	38,844	38,844
Number of gaps in scaffolds	30	39
Total gap length in scaffolds	6000	7800
Average gap length in scaffolds	200	200
Gap N50 in scaffolds	200	200
Gap auN in scaffolds	200	200
Gap L50 in scaffolds	15	20
Largest gap in scaffolds	200	200
Smallest gap in scaffolds	200	200
Base composition (A:C:G:T)	752,087,122:541,204,141:541,131,424:751,838,440	752,018,420:541,573,618:540,761,947:751,907,142
GC content (%)	41.85	41.85

**Table 3:** Chromosomal pseudomolecules in the genome assembly of *Mustela lutreola*, mMusLut2 (autosomes numbered in descending order of size).

Molecule Name	GenBank Sequence	Size (bp)	GC-Content (%)	Unlocalized Count	Merqury’s Quality Value	Merqury’s Error Rate
Chromosome 1	CM059626.1	290,104,894	40.0	0	65.9388	2.5475 × 10^−7^
Chromosome 2	CM059627.1	225,180,311	40.5	0	64.2117	3.7917 × 10^−7^
Chromosome 3	CM059628.1	211,291,007	39.5	0	64.7304	3.3648 × 10^−7^
Chromosome 4	CM059629.1	202,574,368	41.5	1	64.7407	3.3568 × 10^−7^
Chromosome 5	CM059630.1	177,472,721	41.0	1	63.785	4.1831 × 10^−7^
Chromosome 6	CM059631.1	160,010,566	40.5	0	65.6828	2.7022 × 10^−7^
Chromosome 7	CM059632.1	154,078,643	42.0	0	63.1434	4.8491 × 10^−7^
Chromosome 8	CM059633.1	151,511,187	41.5	0	63.7556	4.2116 × 10^−7^
Chromosome 9	CM059634.1	146,271,530	42.5	0	65.0556	3.1221 × 10^−7^
Chromosome 10	CM059635.1	123,813,674	43.0	0	64.5547	3.5038 × 10^−7^
Chromosome 11	CM059636.1	104,548,976	43.5	0	63.8729	4.0993 × 10^−7^
Chromosome 12	CM059637.1	100,611,554	42.5	0	64.2944	3.7201 × 10^−7^
Chromosome 13	CM059638.1	92,103,190	39.5	0	63.4847	4.4826 × 10^−7^
Chromosome 14	CM059639.1	75,872,633	43.0	0	62.7699	5.2846 × 10^−7^
Chromosome 15	CM059640.1	70,922,371	47.0	0	63.3164	4.6598 × 10^−7^
Chromosome 16	CM059641.1	64,033,846	46.0	1	63.6445	4.3207 × 10^−7^
Chromosome 17	CM059642.1	49,398,020	48.5	0	65.3592	2.9113 × 10^−7^
Chromosome 18	CM059643.1	42,373,272	40.5	0	64.4825	3.5625 × 10^−7^
Chromosome X	CM059644.1	133,082,756	40.0	0	66.1191	2.4439 × 10^−7^
Chromosome Y	CM059645.1	6,758,561	47.5	1	65.6101	2.7479 × 10^−7^
Mitochondrion MT	CM059646.1	16,552	39.5	0	-	-
Unplaced		38,844	-	-	-	-

**Table 4:** Mustelids’ genome assembly information from assemblies in the NCBI Genome Assembly database (https://www.ncbi.nlm.nih.gov/datasets/genome/?taxon=9655 (accessed on 24 July 2023) as of July 2023.

Species	Assembly Size (Gbp) ^1^	Assembly Level ^2^	Number of Assembled Chromosomes	Number of Assemblies	Assembly Release Date ^3^	Reference Genome
*Eira barbara*	2.4676–2.4697	S	-	2	05.10.2021	✓
*Enhydra lutris kenyoni*	2.4553	S	-	1	11.09.2017	✓
*Enhydra lutris nereid*	2.4256	S	-	1	24.06.2019	-
*Gulo gulo*	2.4232	S	-	1	06.12.2018	-
*Gulo gulo luscus*	2.2495–2.3882	S	-	3	04.08.2022	✓
*Lontra canadensis*	2.4057	S	-	1	27.01.2020	✓
*Lutra lutra*	1.0815–2.4384	C, Ch	20	2	09.12.2019	✓
*Martes flavigula*	2.4487	Ch	21	1	27.03.2023	✓
*Martes zibellina*	2.4207	S	-	1	20.04.2020	✓
*Meles meles*	2.6927–2.7387	S, Ch	23	2	18.12.2021	✓
*Mellivora capensis*	3.0912	S	-	1	15.01.2019	✓
*Mustela erminea*	1.6402–2.4452	C, Ch	23	2	03.01.2020	✓
***Mustela lutreola***	**1.7870–2.5862**	**Ch**	**21**	**2**	**11.07.2023**	**✓**
*Mustela nigripes*	2.4985	Ch	19	1	24.02.2022	✓
*Mustela nivalis*	2.5010–2.5012	S	-	2	06.07.2021	✓
*Mustela putorius*	2.4566–2.9416	S	-	10	07.08.2019	-
*Mustela putorius furo*	1.0954–2.5771	C, S	-	15	02.06.2011	✓
*Neogale vison*	2.4472–2.6812	S, Ch	15	2	02.01.2018	✓
*Pteronura brasiliensis*	2.6023	S	-	1	15.01.2019	✓
*Taxidea taxus jeffersonii*	2.4160	S	-	1	30.10.2018	✓

^1^ for multiple assemblies, the size of the smallest and largest assembly is indicated; ^2^ C—contig, S—scaffold, Ch—chromosome; ^3^ release date of earliest assembly; newly sequenced genome of the European mink is in bold.

**Table 5:** Sampled individual characteristics.

Feature	Individual	
Assembly identifier	mMusLut2	mMustLut3
Ex-situ Programme (EEP) studbook number	3587	3708
Specimen	M1207	M1287
Sex	♂	♂
Age at sampling (months)	22	9
Site of birth	the Zoological Garden in Osnabrück (Lower Saxony, Germany)	the Zoological Garden in Mönchengladbach (North Rhine-Westphalia, Germany)
Degree of kinship	father (P)	son (F1)
Research use of sample	HiFi (high-fidelity) reads, genome assembly	Hi-C (all-versus-all chromatin conformation capture) data

**Table 6:** European mink genome accessions.

Information	Individual		
General identifiers			
Isolate (assembly identifier)	mMusLut2		mMustLut3
Assembly type	principal pseudohaplotype	alternate pseudohaplotype	-
Whole genome sequencing project accession data			
BioProject	PRJNA984926	PRJNA984927	-
BioSample ID	SAMN35784236		-
WGS project	JAUCGO01	JAUCGP01	-
Raw data accessions			
PacBio Sequel IIe HiFi data	https://genomeark.s3.amazonaws.com/index.html?prefix=species/Mustela_lutreola/mMusLut2/ (accesed on 1 July 2023)		-
Hi-C Dovetail Genomics data	-	-	https://genomeark.s3.amazonaws.com/index.html?prefix=species/Mustela_lutreola/mMusLut3/ (accesed on 1 July 2023)
Genome assembly			
GenBank Accession	GCA_030435805.1	GCA_030435785.1	-
Mitochondrial Assembly (GenBank)	CM059646.1		-

## Data Availability

The metadata for spectral estimates, sequencing runs, contaminants, and pre-curation and curated assembly statistics that support the findings of this study are available in the GenomeArk repository with the identifier “*Mustela lutreola*” (https://genomeark.github.io/genomeark-all/Mustela_lutreola.html (accesed on 1 July 2023) and https://genomeark.s3.amazonaws.com/index.html?prefix=species/Mustela_lutreola (accesed on 1 July 2023)).
